# miR-137 alleviates doxorubicin resistance in breast cancer through inhibition of epithelial-mesenchymal transition by targeting DUSP4

**DOI:** 10.1038/s41419-019-2164-2

**Published:** 2019-12-04

**Authors:** Feiya Du, Ling Yu, Ying Wu, Shuqian Wang, Jia Yao, Xiaoxiao Zheng, Shangzhi Xie, Shufeng Zhang, Xuemei Lu, Yu Liu, Wei Chen

**Affiliations:** 1 Department of Orthopaedics, First Affiliated Hospital, School of Medicine, Zhejiang University, Hangzhou, 310003 China; 2 Department of Nephrology, the Children’ s Hospital, Zhejiang University School of Medicine, Hangzhou, China; 3 Cancer Institute of Integrated Traditional Chinese and Western Medicine, Key Laboratory of Cancer Prevention and Therapy Combining Traditional Chinese and Western Medicine, Zhejiang Academy of Traditional Chinese Medicine,Tongde Hospital of Zhejiang province, Hangzhou, Zhejiang 310012 China; 4 Department of General Surgery, The First Affiliated Hospital, School of Medicine, Zhejiang University, 79 Qingchun Road, Hangzhou, 310003 China

**Keywords:** Breast cancer, Cell biology

## Abstract

Acquired resistance to chemotherapy is a major obstacle in breast cancer (BC) treatment. Accumulated evidence has uncovered that microRNAs (miRNAs) are vital regulators of chemoresistance in cancer. Growing studies reveal that miR-137 acts as a suppressor in tumor progression. However, it remains obscure the role of miR-137 in modulating the sensitivity of BC cells to doxorubicin (DOX). In this study, we demonstrate that miR-137 exerts a significant effect on repressing the development of chemoresistance of BC cells in response to DOX via attenuating epithelial-mesenchymal transition (EMT) of tumor cells in vitro and in vivo. MiR-137 overexpression dramatically elevated the sensitivity of BC cells to DOX as well as impaired the DOX-promoted EMT of tumor cells. Mechanistically, miR-137 directly targeted dual-specificity phosphatase 4 (DUSP4) to impact on the EMT and chemoresistance of BC cells upon DOX treatment. Consistently, decreased DUSP4 efficiently enhanced the sensitivity of BC cells to DOX while overexpressed DUSP4 significantly diminished the beneficial effect of miR-137 on BC cells chemoresistance. Moreover, the increased miR-137 heightened the sensitivity of BC cells-derived tumors to DOX through targeting DUSP4 in vivo. Together, our results provide a novel insight into the DOX resistance of BC cells and miR-137 may serve as a new promising therapeutic target for overcoming chemoresistance in BC.

## Introduction

Breast cancer (BC) is one of the most frequently diagnosed cancers in women worldwide^1^. Although surgery may be effective in some early cases, adjuvant chemotherapy is important for improving survival, especially in patients with end-stage disease^2,3^. Doxorubicin (DOX) and its analog epirubicin are widely used in most chemotherapeutic regimens in BC^4^. However, intrinsic or acquired resistance to DOX limits the clinical outcomes of DOX-based regimens^5^. The potential mechanisms underlying DOX resistance remain to be explored.

Epithelial-mesenchymal transition (EMT) is the biological process by which epithelial cells are transformed into mesenchymal-phenotype cells. EMT plays a vital role in embryogenesis, wound healing, and tumor pathogenesis^6,7^. Highly active EMT is often detected in cancer progression, and the abnormally activated EMT enables cells to acquire highly malignant properties, including mobility, invasiveness, and distant metastasis ability^8,9^. Moreover, an increasing number of studies have indicated that cancer cells that undergo EMT acquire cancer stem cell characteristics, significantly promoting the development of chemoresistance^10^. In fact, targeting EMT can overcome chemoresistance and suppress tumor metastasis^6^. In BC, accumulating evidence has demonstrated that EMT is involved in drug resistance.

MicroRNAs (miRNAs) are a recently discovered class of non-coding RNAs that play key roles in regulating gene expression at post-transcriptional level by binding the 3′ untranslated region (3′-UTR) of mRNA. Aberrant expression of miRNAs contributes to the overactivation of specific oncogenes, enhancing cancer development^11,12^. Abnormal miRNA expression has also been identified in BC and is closely correlated with proliferation, invasion, metastasis, and chemoresistance^13,14^. miR-137 is frequently downregulated in some solid tumors, including colon cancer^15^, non-small cell lung cancer^16^, pancreatic cancer^17^, gastric cancer^18^, and osteosarcoma^19^, and potentially acts as a tumor suppressor in these tumors. Though miR-137 is involved in chemoresistance in several cancers^20–22^, its physiological role in DOX resistance of BC is not well elucidated.

In the present study, we demonstrate that overexpressed miR-137 alleviated the development of DOX resistance in BC cells and dual-specificity phosphatase 4 (*DUSP4*) is a novel target gene of miR-137. Though targeting *DUSP4*, miR-137 attenuated the EMT of BC cells in the setting of DOX treatment.

## Results

### miR-137 played a role in DOX chemoresistance of BC cells

**Fig. 1:**
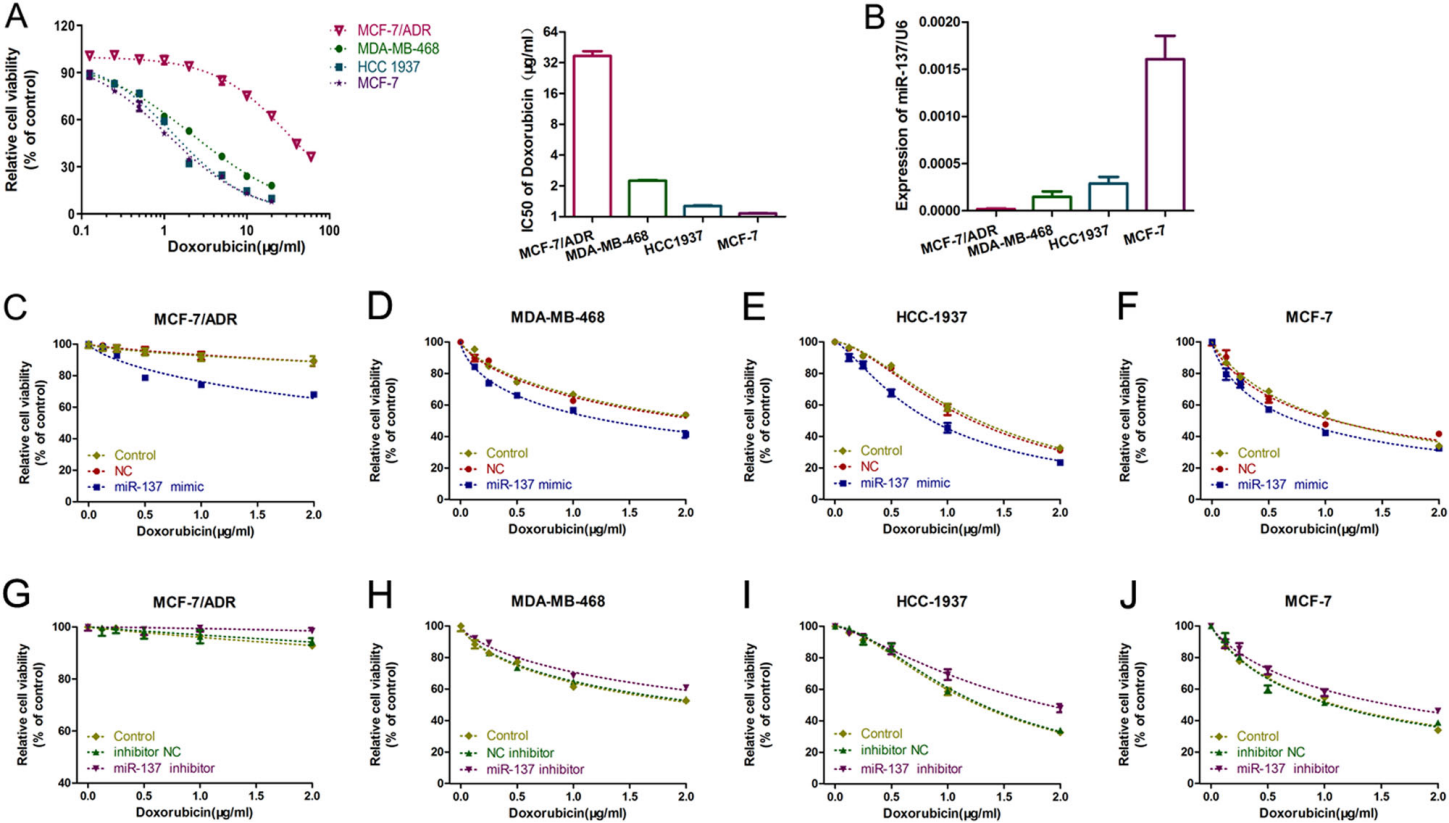
miR-137 overexpression sensitized BC cells to DOX. **a** CCK-8 showing viability of BC cell lines under DOX treatment (0, 0.3125, 0.625, 1.25, 2.5, 5, 10, 20 μg/ml) for 48 h; the IC50 value was calculated based on the CCK-8 results. **b** Detection of miR-137 expression in BC cells; the result was quantified by comparing with the internal control U6. **c**–**g** Detection of viability of BC cells transfected with miR-137 mimics (5 nM) or negative control (NC) RNA and cultured with 0, 0.5, 1.0, 1.5, and 2.0 µg/ml DOX for 48 h. **h**–**l** Detection of viability of BC cells transfected with miR-137 inhibitor (5 nM) or NC RNA and cultured with 0, 0.5, 1.0, 1.5, and 2.0 µg/ml DOX for 48 h. All data are representative of three independent experiments.

To investigate the biological role of miR-137 in DOX resistance of BC, we first studied the viability of four BC cell lines (MCF-7/ADR [adriamycin-resistant], MDA-MB-468, HCC1937, MCF-7) following exposure to different concentrations of DOX. Upon DOX treatment, MCF-7/ADR cell line displayed the highest levels of viability and median inhibitory concentration (IC50) while MCF-7 cell line showed the lowest (Fig. 1a). Then, we evaluated the expression levels of miR-137 in the four BC cell lines. Surprisingly, the levels of miR-137 expression exhibited an opposite trend relative to that of cell viability and IC50 value (Fig. 1b), indicating a positive correlation between miR-137 expression and DOX sensitivity in BC cells.

To further explore the function of miR-137 in chemoresistance to DOX, miR-137 expression in BC cell lines were manipulated using miR-137 mimics and inhibitor. After transfected with miR-137 mimics or inhibitor, the viability of the tumor cells was evaluated in the presence of DOX. miR-137 overexpression robustly amplified the inhibitory effects of DOX on BC cells (Fig. 1c–g), while decreased miR-137 significantly enhanced the resistance to DOX of BC cells (Fig. 1h–l). Consistently, IC50 of DOX in BC cells was also reduced after miR-137 overexpression while increased by miR-137 abrogation (Fig. S1). These results indicate a vital role for miR-137 in repressing the development of DOX resistance in BC cells.

### miR-137 inhibited DOX-induced EMT in BC cells

**Fig. 2:**
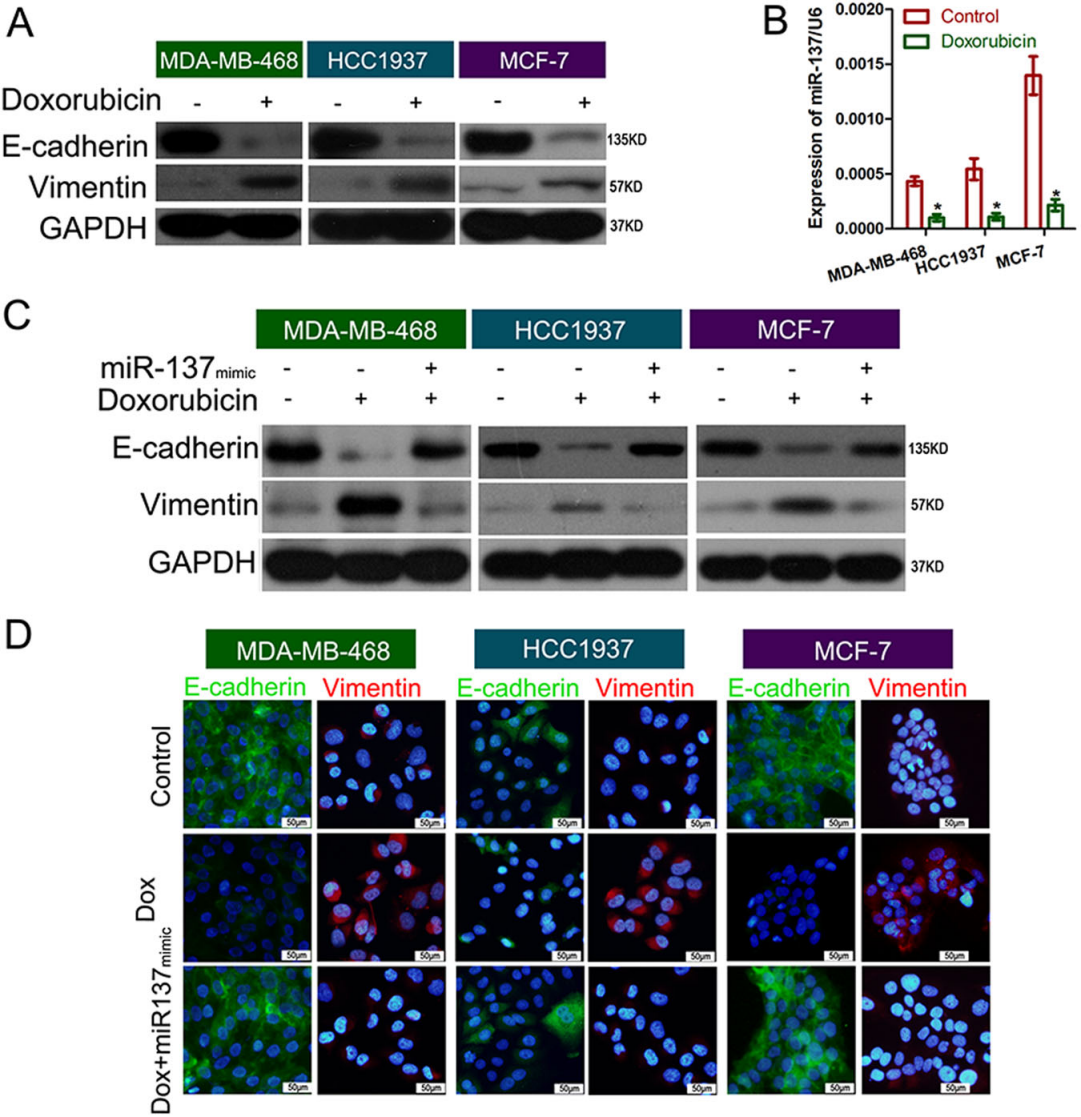
miR-137 overexpression inhibited DOX-induced EMT in BC cells. **a** Effect of DOX (IC50) on E-cadherin and vimentin expression in BC cell lines determined by western blotting. **b** Effect of DOX (IC50) on miR-137 expression in BC cell lines detected by qPCR. **c** Western blotting detection of E-cadherin and vimentin expression levels in BC cells treated with control, DOX, or DOX plus miR-137 mimics. **d** Immunofluorescence detection of E-cadherin and vimentin expression in BC cells treated with control, DOX, or DOX plus miR-137 mimics. *P *<* 0.05 versus control. All data are representative of three independent experiments.

As acting an important role in chemoresistance in BC, EMT of tumor cells were explored to determine whether EMT is involved in DOX sensitivity mediated by miR-137 in BC cells. We first tested the expression of the epidermal marker E-cadherin, the mesenchymal marker Vimentin, and miR-137 in three BC cell lines (MDA-MB-468, HCC1937, MCF-7) when treated with DOX at a concentration of their respective IC50. The treatment of DOX significantly increased Vimentin protein levels and decreased E-cadherin levels in three cell lines, suggesting the induction of EMT in BC cells (Fig. 2a). Meanwhile, the levels of miR-137 were obviously reduced in the three cell lines upon the DOX treatment (Fig. 2b). Next, BC cells were transfected with miR-137 mimics and then subjected to DOX treatment. We found that overexpressed miR-137 efficiently reversed the EMT of BC cells which was induced by DOX (Fig. 2c). Furthermore, the similar changes of EMT-associated proteins E-cadherin and Vimentin were observed in BC cells by immunofluorescence staining (Fig. 2d). These findings reveal that miR‐137 act as a repressor in regulating DOX-induced EMT in BC cells.

### DUSP4 is a direct target of miR‐137

**Fig. 3:**
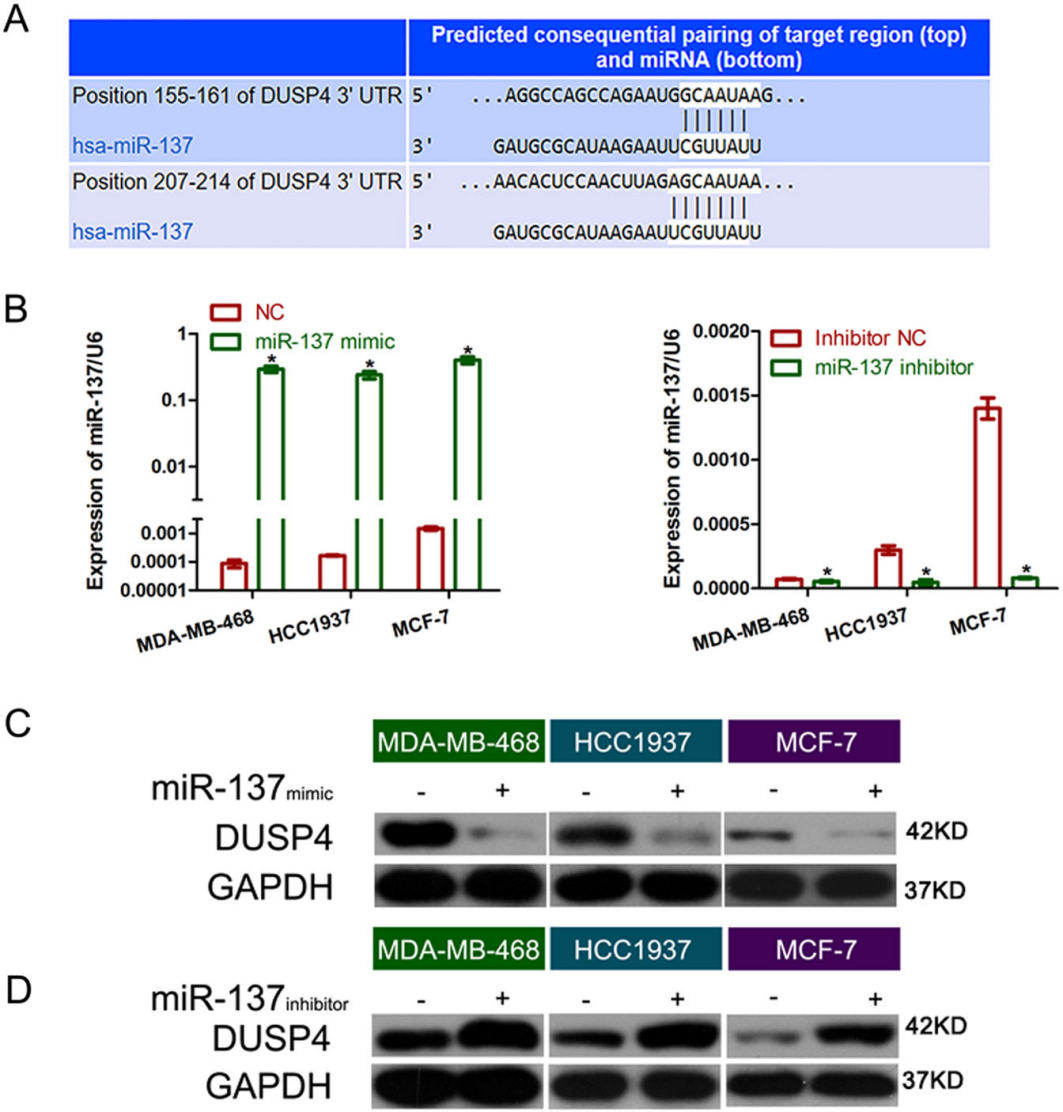
miR-137 regulated DUSP4 negatively. **a** TargetScan-predicted binding sequences of miR-137 in the 3′-UTR of *DUSP4*. **b** Quantification of *DUSP4* mRNA expression in BC cells transfected with miR-137 mimics, miR-137 inhibitor, or NC. **c**, **d** Quantification of DUSP4 protein expression in BC cells transfected with miR-137 mimics, miR-137 inhibitor, or NC. All data are representative of three independent experiments.

To investigate the detailed mechanism underlying miR‐137-regulating DOX resistance of BC cells, we used a miRNA target prediction website (targetscan.org) to screen for miR‐137 target genes, and identified *DUSP4*, which contains the binding sequence of miR‐137, as a tentative target of miR‐137 (Fig. 3a). To investigate the association between miR‐137 and *DUSP4* expression, we explored *DUSP4* expression levels in BC cell lines with miR‐137 overexpression or inhibition. Both mRNA and protein levels of *DUSP4* were significantly decreased in miR‐137-overexpressing cells compared to that of the control cells (Fig. 3b, c). In contrast, inhibition of miR‐137 increased DUSP4 expression at both mRNA and protein levels (Fig. 3b, d).

### DUSP4 mediated the effect of miR‐137 on regulating DOX resistance and EMT

**Fig. 4:**
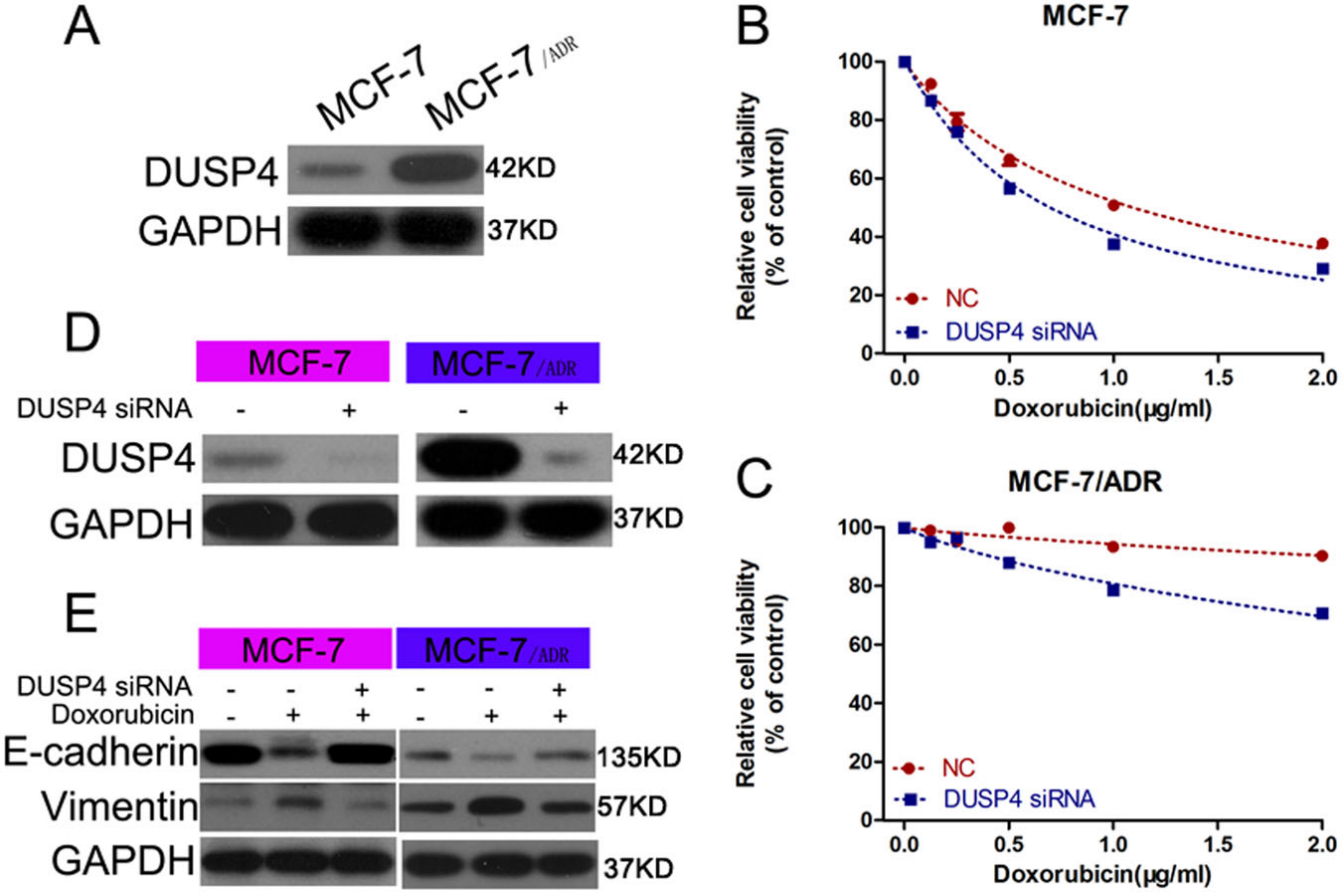
DUSP4 knockdown inhibited DOX-induced EMT in BC cells. **a** DUSP4 protein levels in MCF-7 and MCF-7/ADR cells. **b**, **c** Detection of viability of MCF-7 and MCF-7/ADR cells transfected with DUSP4 siRNA or NC and cultured with 0, 0.5, 1.0, 1.5, and 2.0 µg/ml DOX. **d** Western blotting confirmation of RNA interference efficiency of DUSP4 siRNA. **e** Western blotting detection of E-cadherin and vimentin expression levels in BC cells treated with control, DOX, or DOX plus DUSP4 siRNA. All data are representative of three independent experiments.

To identify the association between DUSP4 and miR-137 in BC, we tested protein levels of DUSP4 in MCF-7 and MCF-7/ADR cells. Relative to that of MCF-7 cells, DUSP4 was higher in the MCF-7/ADR cells (Fig. 4a). The results of Cell Counting Kit-8 (CCK-8) assays showed that the abrogation of DUSP4 sensitized BC cells to DOX (Fig. 4b, c), and the results of western blotting confirmed the RNA interference efficiency of small interfering RNA (siRNA) targeting DUSP4 (Fig. 4d). Finally, we investigated the role of DUSP4 in regulating DOX-mediated EMT. Similar to that of miR-137 overexpression, DUSP4 knockdown reversed DOX-mediated EMT of BC cells (Fig. 4e). These results indicate that DUSP4 may be involved in the effect of miR-137 on BC cells.

**Fig. 5:**
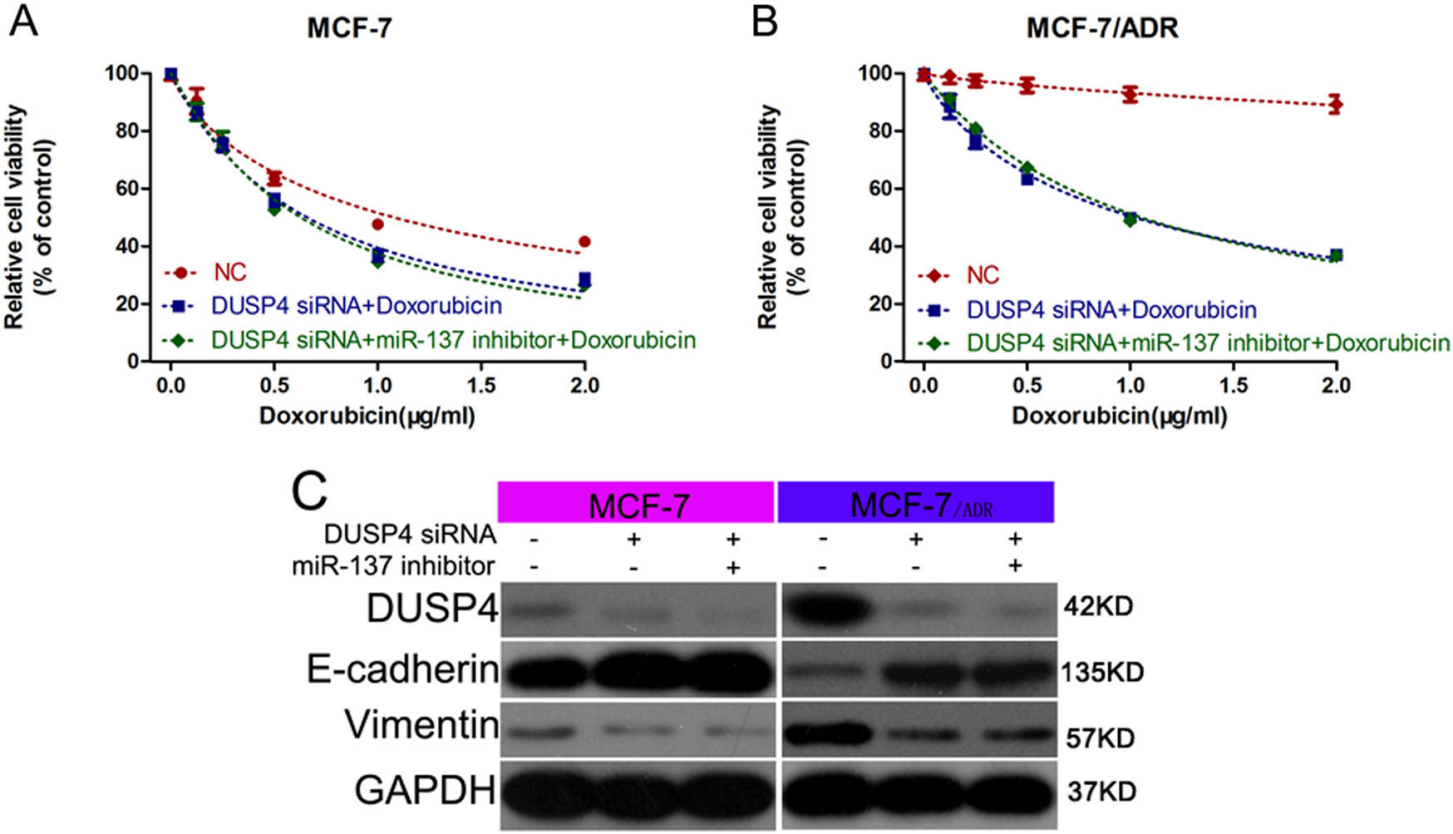
DUSP4 knockdown eliminated miR-137 inhibitor-mediated regulation of DOX sensitivity and EMT. **a**, **b** CCK-8 detection of viability of MCF-7 and MCF-7/ADR cells transfected with DUSP4 siRNA alone or with both DUSP4 siRNA and miR-137 inhibitor and cultured with 0, 0.5, 1.0, 1.5, and 2.0 µg/ml DOX. **c** Western blotting detection of E-cadherin and vimentin expression in MCF-7 and MCF-7/ADR cells transfected with DUSP4 siRNA alone or with both DUSP4 siRNA and miR-137 inhibitor. All data are representative of three independent experiments.

**Fig. 6:**
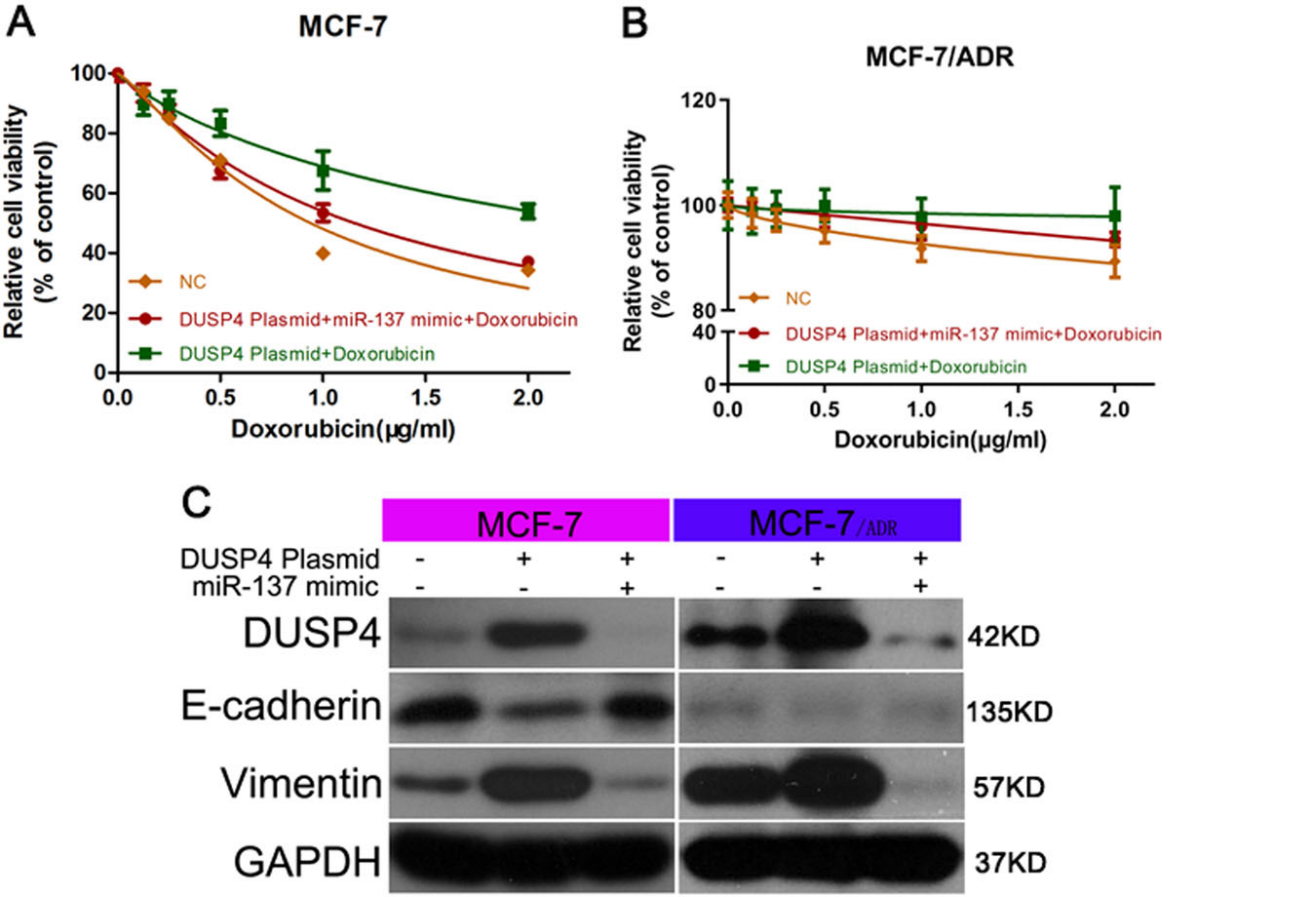
DUSP4 overexpression reversed miR-137 mimic-mediated regulation of DOX sensitivity and EMT. **a**, **b** CCK-8 detection of viability of MCF-7 and MCF-7/ADR cells transfected with DUSP4 vector or with both DUSP4 vector and miR-137 mimic and cultured with 0, 0.5, 1.0, 1.5, and 2.0 µg/ml DOX. **c** Western blotting detection of E-cadherin and vimentin expression in MCF-7 and MCF-7/ADR cells transfected with DUSP4 vector or with both DUSP4 vector and miR-137 mimic. All data are representative of three independent experiments.

To confirm this, we performed rescue experiments by transfecting DOX-treated MCF-7 and MCF-7/ADR cells with DUSP4 siRNA combined with miR-137 inhibitor, or DUSP4 overexpression vector combined with miR-137 mimics. The results of CCK-8 assays showed that DUSP4 knockdown disrupt the miR-137 inhibition-mediated DOX resistance when compared with that of the control group (Fig. 5a, b). The results of western blotting showed no differences of E-cadherin and Vimentin expression between the DUSP4 siRNA group and DUSP4 siRNA plus miR-137 inhibitor group (Fig. 5c), which demonstrated that DUSP4 is involved in miR-137-regulating EMT in the presence of DOX. These results were confirmed by the data from DUSP4 and miR-137 co-overexpression experiments (Fig. 6).

### miR‐137 overexpression promoted DOX sensitivity in vivo

**Fig. 7:**
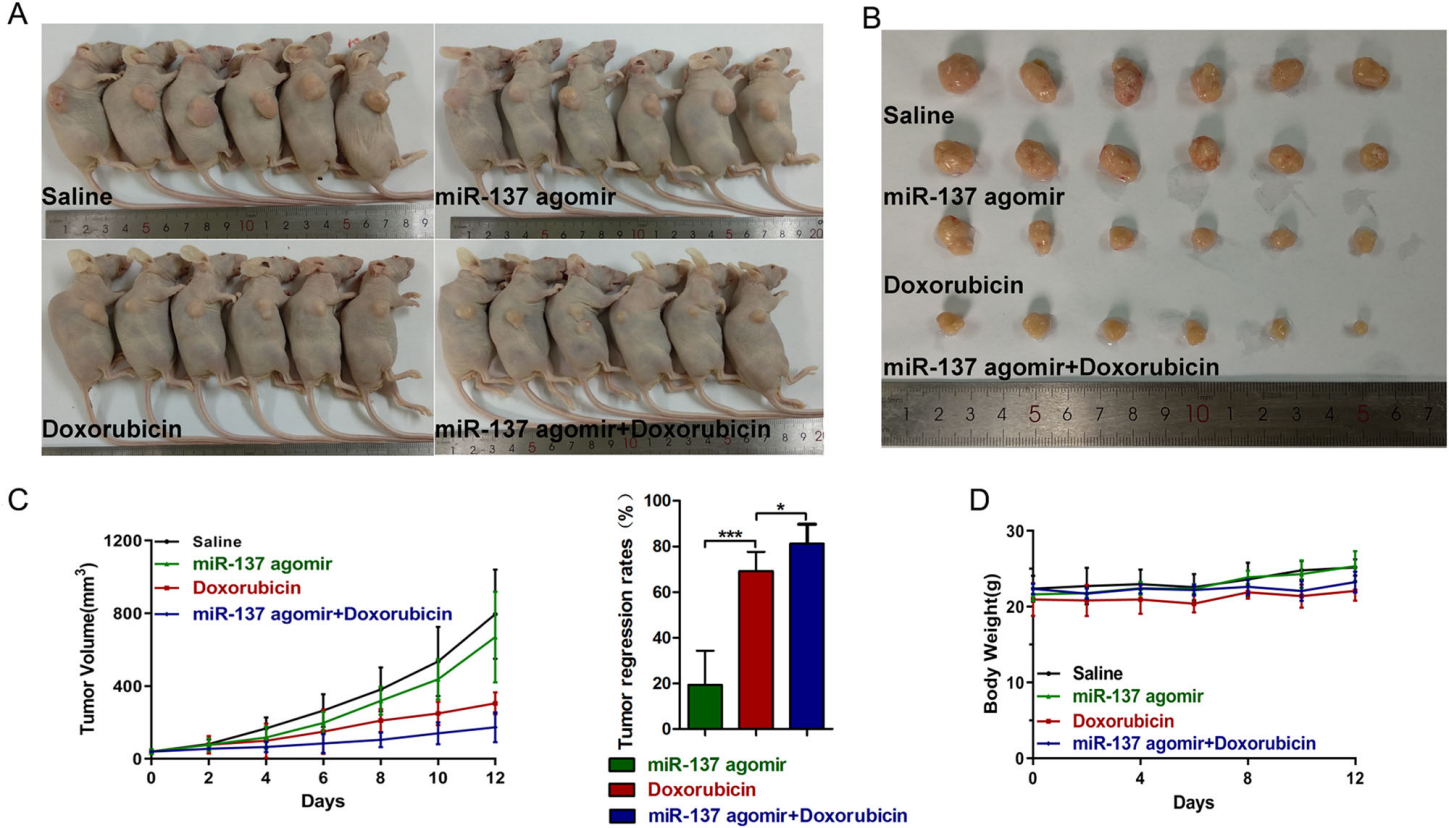
miR-137 overexpression enhanced the anti-BC activity of DOX in vivo. **a**, **b** Representative tumor images (*n* = 6 mice per group). **c** Tumor volumes were measured every other day. The tumor regression rate was calculated based on the tumor volumes. *P < 0.05, **P < 0.01, ***P < 0.001. **d** The mouse body weight in each group was measured every other day.

To explore the role of miR-137 in DOX resistance in vivo, we confirmed the results of in vitro experiments using a mouse xenograft model. The animal experiment results clearly showed that, in the MCF-7 xenograft model, the treatment of miR‐137 agomir combined with DOX exhibited more dramatical inhibition on tumor growth than vehicle control or single-drug treatment (Fig. 7). Immunohistochemistry analyses revealed marked reduction of Ki-67-positive cells in combination treatment group (Fig. S2). Consistently, the results of terminal deoxyribonucleotidyl transferase (TDT)-mediated dUTP-digoxigenin nick end labeling (TUNEL) assays revealed that combination treatment group displayed significantly increased amount of apoptotic cells (Fig. S2). Additionally, immunohistochemistry analyses also showed that miR-137 agomir treatment reduced the DUSP4 expression (Fig. S2). The efficiency of miR-137 agomir in the xenograft model tumor tissues was quantified by qPCR (Fig. S3). Taken together, these findings show that miR‐137 correlates negatively with DOX resistance in BC and that miR‐137 overexpression may be a useful strategy for enhancing chemosensitivity in the treatment of BC.

## Discussion

Although the application of chemotherapy drugs and the development of chemotherapy regimens have significantly improved the survival rate of patients with BC, the development of multi-drug resistance (MDR) remains one of the main reasons for treatment failure^23,24^. miRNAs inhibit gene expression by inhibiting transcription or inducing degradation of the targeted gene^25^. Much evidence shows that miRNA expression is frequently abnormal in BC and that miRNAs participate in chemoresistance of BC^26,27^. However, the miRNA regulatory network is quite complex, and miRNA biological roles in chemoresistance of BC have not been fully elucidated.

In cancer, miR-137 is often downregulated due to hypermethylation of its CpG island^28^. miR-137 hypermethylation leads to its inhibition in many tumors, such as colorectal cancer, pancreatic cancer, and endometrial cancer^29–31^. Functional analysis has indicated that miR-137 can inhibit cell proliferation, cell cycle arrest and apoptosis, cell migration and invasion, and affect chemoresistance^32–36^. For example, miR-137 overexpression sensitized resistant colon cancer cells to oxaliplatin^20^. In hepatocellular carcinoma, miR-137 upregulation reverses sorafenib resistance and cancer-initiating cell phenotypes by degrading solute carrier family 25 member 5 (ANT2)^21^. Zhu et al. reported that miR-137 was involved in MDR in BC through modulation of P-glycoprotein (P-gp) by targeting Y-box binding protein 1 (YB-1)^37^. Cheng et al. have reported that the miR-137-FSTL1-integrin beta 3-Wnt-β-catenin signaling axis in BC cells can regulate stemness and chemoresistance^38^. In the present study, we confirmed that miR-137 is involved in DOX resistance of BC.

Here, we also clarify the EMT inhibitory mechanism of miR‐137 in BC. EMT occurs when epithelial cells lose contact with the environment and present mesenchymal phenotypes, promoting their entry into the bloodstream and migration to distant locations. BC is an aggressive cancer with high EMT-related metastasis^39^. Interestingly, recent studies have shown that EMT is also closely related to the development of chemoresistance and that inhibiting EMT can eliminate chemoresistance in BC^40^. EMT is an important therapeutic target in BC metastasis and chemotherapy resistance in BC^41^. In the present study, we found for the first time that miR‐137 regulates EMT negatively to improve DOX sensitivity in BC cells.

Bioinformatics analysis identified *DUSP4* as a novel target of miR‐137 in BC cells. Herein, our data show that restoring miR‐137 expression in BC cells led to the suppression of *DUSP4* expression. DUSP4 upregulation has been reported in relation to drug resistance and patient outcome in BC^42–44^. Silencing *DUSP4* significantly sensitized BC cells to trastuzumab^45^. *DUSP4* knockdown reversed EMT in MCF-7/ADR cells and increased DOX chemosensitivity of MCF-7/ADR cells^46^. These findings suggest that *DUSP4* is an important gene that modulates MDR in BC cells. Our results confirm that miR-137 can directly target the 3′-UTR of *DUSP4* mRNA and thereby decrease its expression in BC cells. We also observed that both miR-137 overexpression and DUSP4 knockdown significantly suppressed EMT and reduced DOX resistance. Based on these findings, we infer that *DUSP4* is a functional target gene of miR-137, regulating DOX resistance in BC cells.

In conclusion, we have identified a novel mechanism underlying miR‐137 modulated DOX resistance in BC. Restoration of miR‐137 promotes the DOX sensitivity of BC cells through EMT inhibition by targeting *DUSP4*. Our findings may help to establish new strategies for improving therapeutic options for patients with DOX-resistant BC.

## Materials and methods

### Cell cultures

The MCF-7 human BC cell line was maintained in RPMI 1640 medium supplemented with 10% fetal bovine serum (Invitrogen, Carlsbad, CA, USA). The HCC1937 and MDA‐MB‐468 cells were maintained in Dulbecco’s modified Eagle’s medium (DMEM) containing 10% fetal bovine serum (Invitrogen). All cell lines were obtained from ATCC (Manassas, VA, USA) and were incubated at 37 °C in a humidified atmosphere with 5% CO_2_. For the MCF-7/ADR cells, the resistant variants were originated by growing initial MCF-7 cells with raising concentrations of DOX (Sigma-Aldrich, St. Louis, MO, USA) (from 0.1 to 60 μg/ml). DOX was added every 3 days. Cell viability was analyzed by CCK-8 assay every month. IC50 values of MCF-7 and MCF-7/ADR cells were 1.0 and 35 μg/ml of DOX, respectively. MCF-7/ADR cells were 35 times as much resistant to the cytotoxic effect DOX as compared with the initial MCF-7 cells. DOX was added at a final concentration of 1 mg/l to maintain the resistance phenotype until 1 week prior to the experiments.

### Cell transfection

MiR-137 mimic, miR-137 inhibitor or negative control (NC) were synthesized by GenePharma (Shanghai, China). DUSP4 siRNA and negative control were purchased from QIAGEN (Valencia, CA, USA). BC cells were seeded onto a 6-well plate at 2 × 10^5^/well. When the cells reached 50% confluence, they were transfected with miR-137 mimic (5 nM), miR-137 inhibitor (5 nM), DUSP4 siRNA (5 nM) or negative control (5 nM). Cell transfections were performed using Lipofectamine 2000 Reagents (Invitrogen, Thermo, IL, USA) according to the manufacturer’s instructions. After 48 h transfection, the cells were used for subsequent experiments. The sequences of miR-37 mimics/inhibitor, DUSP4 siRNA were followed:

mir-137-3p mimics: 5’-TTATTGCTTAAGAATACGCGTAG-3’ and 5’-ACGCGTATTCTTAAGCAATAATT-3’

mir-137-3p inhibitor: 5’-CTACGCGTATTCTTAAGCAATAA-3’

DUSP4 siRNA:

DUSP4-homo-1172

sense: 5'- CCACUUUGAAGGACACUAUTT-3'

antisense: 5'-AUAGUGUCCUUCAAAGUGGTT-3'

DUSP4-homo-929

sense: 5'- GUACCCAGAAUUCUGUUCUTT-3'

antisense: 5'-AGAACAGAAUUCUGGGUACTT-3'

DUSP4-homo-1416

sense: 5'-GCAUCAUCUCGCCCAACUUTT-3'

antisense: 5'-AAGUUGGGCGAGAUGAUGCTT-3'

### Cell viability assay

Cell viability was detected using CCK-8 (CK04, Dojindo, Tokyo, Japan). Briefly, 4000 cells were seeded into each well of 96-well plates and then maintained overnight. After treatment with a series of concentration of doxorubicin (0, 0.125, 0.25, 0.5, 1, and 2 μg/ml) for 48 h, cells were incubated with CCK-8 reagent for 2 h, followed by optical density reading at 450 nm. For the analysis of miR-137 effect on cell viability, BC cells were transfected with miR-137 inhibitor, miR-137 mimic or negative control. Then cells or transfected cells were exposed to different concentration of doxorubicin for 48 h, and then cell viability was evaluated.

### qPCR

Total RNA from the cells subjected to different treatments was isolated using TRIzol (Invitrogen, Thermo Fisher Scientific, Rockford, IL, USA). For miR-137 detection, cDNA was synthesized from total RNA using a miScript II RT Kit (QIAGEN, Shanghai, China). qPCR was performed using a miScript SYBR Green PCR Kit (QIAGEN), using *U6* as a normalization gene. The qPCR was performed on the 7300 Real Time PCR System (Applied Biosystems, Foster City, CA, USA). Data was calculated by 2−ΔΔCt method. The following primers were used: mir-137-3p: 5′-TTATTGCTTAAGAATACGCGTAG-3′

### Western blotting

The cells subjected to different treatments were harvested in radioimmunoprecipitation assay lysis buffer (Beyotime, Shanghai, China), and the protein concentration was measured using the bicinchoninic acid protein assay (Thermo Fisher Scientific). Proteins were resolved by 10% sodium dodecyl sulfate-polyacrylamide gel electrophoresis, transferred onto polyvinylidene fluoride membranes, blocked with 5% skimmed milk in Tris-buffered saline, and reacted with primary antibodies against E-cadherin (1:3000; ab1416, Abcam, Cambridge, MA, USA), vimentin (1:5000; #5741, CST, Danvers, MA, USA), DUSP4 (1:2000; ab72593, Abcam), and glyceraldehyde-3-phosphate dehydrogenase (GAPDH, 1:2000; ab9485, Abcam). GAPDH was used as an internal control. Thereafter, the membranes were washed with TBST and further incubated with a horseradish peroxidase-conjugated secondary antibody (#7074, #7076, CST) at a 1:2000 dilution for 2 h at room temperature. The bands were visualized using ECL solution (Pierce).

### Immunofluorescence

BC cells were seeded in glass slides at a density of 0.5–1 × 10^5^ cells. After 24 h treatment, the cells were fixed with 4% paraformaldehyde for 15 min and then incubated with fluorescein isothiocyanate (FITC)-conjugated primary antibodies against E-cadherin (1:100, ab1416, Abcam) and vimentin (1:50, #5741, CST) overnight at 4 °C. The nuclei were stained with 4’6-diamidino-2-phenylindole (DAPI). Confocal fluorescence microscopy was used to observe and photograph fluorescent sections.

### Animal experiments

For the subcutaneous tumor growth assay, 2 × 10^6^ cells (MCF-7) suspended in 0.1 ml phosphate-buffered saline (PBS) were subcutaneously injected into the dorsal flank of per male BALB/c nude mouse (~6 weeks old). Tumor diameter in nude mice was daily measured by digital calipers. After tumors reaching 0.5 cm in diameter, the mice were randomly divided in to four groups (*n* = 6 mice per group): PBS, DOX, miR-137 agomir, or DOX in combination with miR-137 agomir. MiR-137 agomir was chemically modified from miR-137 mimic (RiboBio, Guangzhou, China). For the treatment, the mice of the four groups received multi-point intratumoral injections of miR-137 agomir (2 nmol per mouse, every 3 days), miR-137 agomir (2 nmol per mouse every 3 days) in combination with DOX (2 mg/kg body weight, every 2 days), DOX (2 mg/kg body weight, every 2 days), or control (normal saline, every 2 days), respectively. Tumor volume was calculated by the formula 0.5 × length × width^2^ (mm^3^). All animal care and experimentation were conducted according to the guidelines of the Institutional Animal Care and Use Committee of the First Affiliated Zhejiang Hospital.

### Immunohistochemistry

Immunohistochemistry was performed to detect Ki-67 and DUSP4 expression in the mouse tumor tissues. Briefly, tumor tissues were fixed in 10% formalin for paraffin block preparation. tumor slices were permeabilized with blocking buffer (5% BSA/0.25% TX-100 in PBS) and incubated with the Ki-67 (1:100, ab15580, Abcam) or DUSP4 (1:50, ab72593, Abcam) antibody overnight at 4 ^o^C. After washing with PBS, samples were incubated with HRP-conjugated secondary antibody (Invitrogen, Carlsbad, CA) before analysis by microscopy (PerkinElmer, Waltham, MA). Ki67- and DUSP4-positive cells were quantified using Image J software.

### TUNEL

TUNEL was performed to detect apoptotic cells in mouse tissue slides using a TUNEL kit according to the manufacturer’s instructions (Thermo Fisher Scientific). In brief, 4-μm-thick paraffin sections were deparaffinized in xylene three times for 5 min and hydrated with different concentration of ethanol. Apoptotic cells were stained with diaminobenzidine (DAB) reaction mixture supplied by the kit. The apoptotic cell nuclei were stained in brown and observed under a light microscope (Olympus, Tokyo, Japan). The positive rates were measured using Image J software.

### Statistical analysis

All data are presented as the mean ± standard deviation (SD), and significant differences between treatment groups were analyzed by Student’s *t*-test or one‐way analysis of variance (ANOVA) and Duncan’s multiple range test using SAS statistical software version 6.12 (SAS Institute, Cary, NC, USA). Statistical significance is defined as *P < 0.05, **P < 0.01, ***P < 0.001.

## Supplementary information

supplementary information

figure S1

figure S2

figure S3
