# Supplementary material for: miR-137 alleviates doxorubicin resistance in breast cancer through inhibition of epithelial-mesenchymal transition by targeting DUSP4
Source: Cell Death Dis. 2019 Dec 4;10(12):922. doi: 10.1038/s41419-019-2164-2 (PMC6892819; doi:10.1038/s41419-019-2164-2)
Supplement: Supplementary file 1 — supplementary information [file 41419_2019_2164_MOESM1_ESM.docx]

**Figure legends**

**Fig. S1 Quantitative diagrams of IC50 after BC cells transfected with mir-137 mimic or inhibior**

**Fig. S2 Immunohistochemical assay and TUNEL of xenograft model tissues.** Representative Ki-67 and DUSP4 immunohistochemical staining images in each group are shown; the positive rate was identified by PPI 6.0 software. Apoptotic cells were visualized with TUNEL.

**Fig. S3 qRT-PCR detection of miR-137 expression in xenograft model tissues.**
