# Supplementary figures and images for: miR-137 alleviates doxorubicin resistance in breast cancer through inhibition of epithelial-mesenchymal transition by targeting DUSP4
Source: Cell Death Dis. 2019 Dec 4;10(12):922. doi: 10.1038/s41419-019-2164-2 (PMC6892819; doi:10.1038/s41419-019-2164-2)

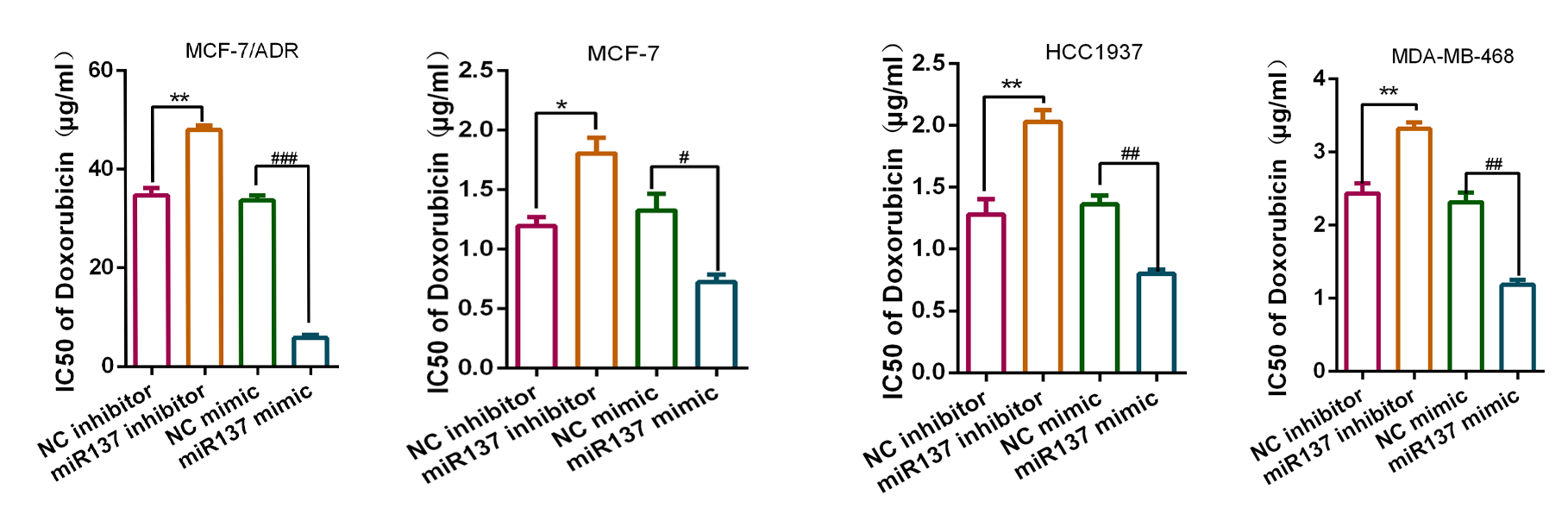

Supplement: Supplementary file 2 — figure S1 [file 41419_2019_2164_MOESM2_ESM.tif]

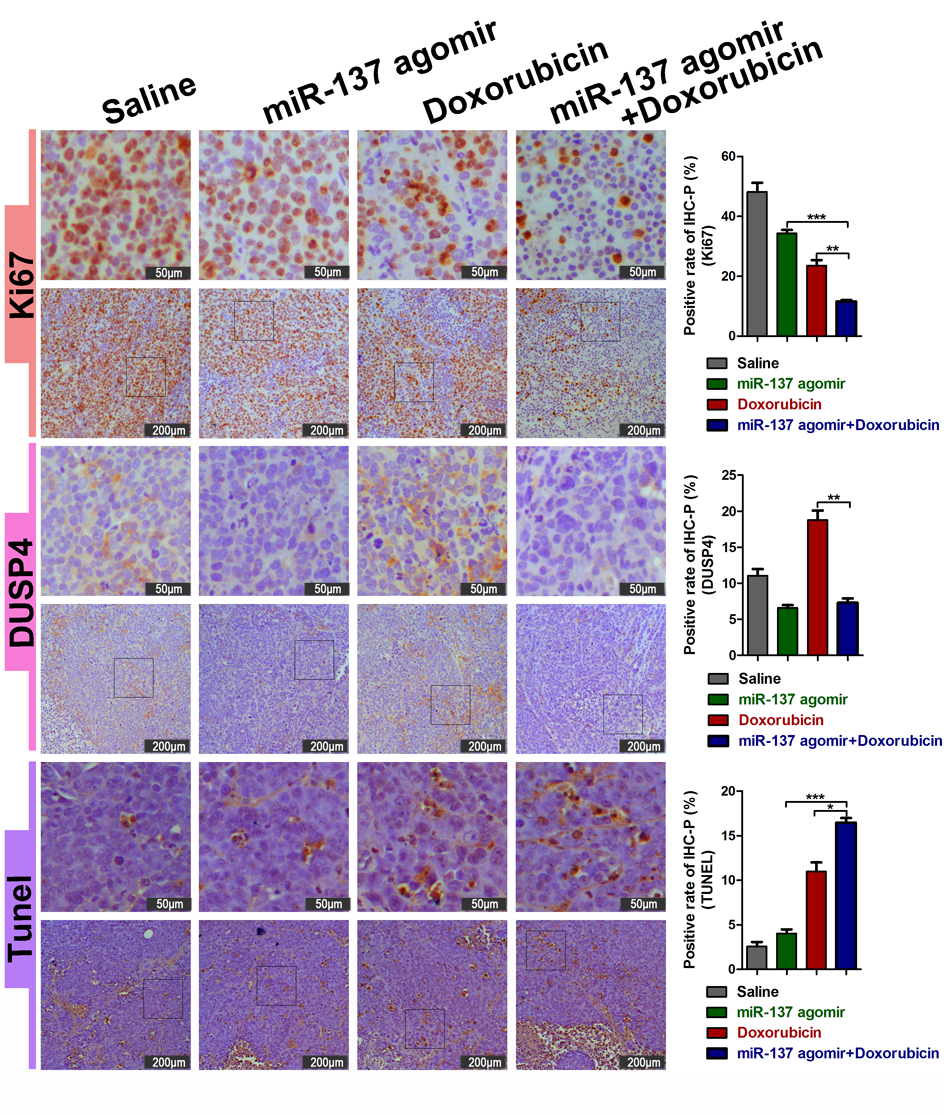

Supplement: Supplementary file 3 — figure S2 [file 41419_2019_2164_MOESM3_ESM.tif]

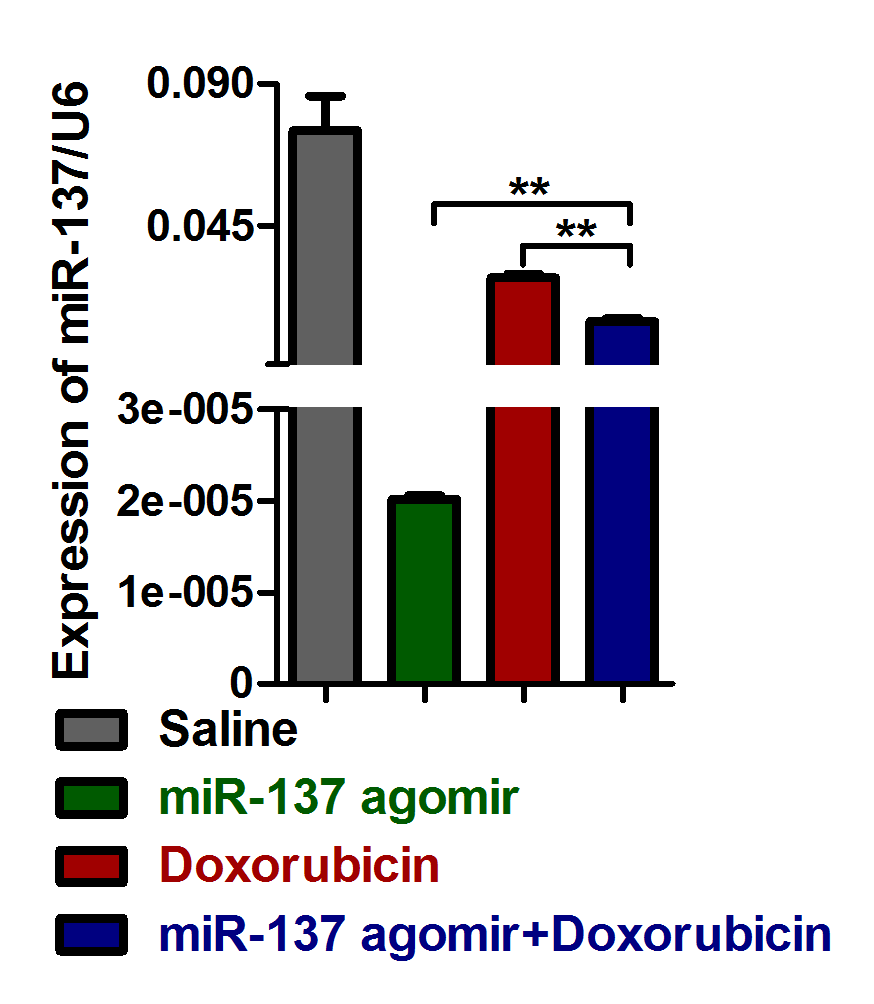

Supplement: Supplementary file 4 — figure S3 [file 41419_2019_2164_MOESM4_ESM.tif]
